# The role of the mass vaccination programme in combating the COVID-19 pandemic: An LSTM-based analysis of COVID-19 confirmed cases

**DOI:** 10.1016/j.heliyon.2023.e14397

**Published:** 2023-03-08

**Authors:** Seng Hansun, Vincent Charles, Tatiana Gherman

**Affiliations:** a Informatics Department, Universitas Multimedia Nusantara, Tangerang, Indonesia; b CENTRUM Católica Graduate Business School, Lima, Peru; c Pontifical Catholic University of Peru, Lima, Peru; d Faculty of Business and Law, University of Northampton, Northampton, NN1 5PH, UK

**Keywords:** Confirmed cases, COVID-19, Deep learning, LSTM, Mass vaccination

## Abstract

The COVID-19 virus has impacted all facets of our lives. As a global response to this threat, vaccination programmes have been initiated and administered in numerous nations. The question remains, however, as to whether mass vaccination programmes result in a decrease in the number of confirmed COVID-19 cases. In this study, we aim to predict the future number of COVID-19 confirmed cases for the top ten countries with the highest number of vaccinations in the world. A well-known Deep Learning method for time series analysis, namely, the Long Short-Term Memory (LSTM) networks, is applied as the prediction method. Using three evaluation metrics, i.e., Mean Absolute Error (MAE), Root Mean Square Error (RMSE), and Mean Absolute Percentage Error (MAPE), we found that the model built by using LSTM networks could give a good prediction of the future number and trend of COVID-19 confirmed cases in the considered countries. Two different scenarios are employed, namely: ‘All Time’, which includes all historical data; and ‘Before Vaccination’, which excludes data collected after the mass vaccination programme began. The average MAPE scores for the ‘All Time’ and ‘Before Vaccination’ scenarios are 5.977% and 10.388%, respectively. Overall, the results show that the mass vaccination programme has a positive impact on decreasing and controlling the spread of the COVID-19 disease in those countries, as evidenced by decreasing future trends after the programme was implemented.

## 1 Introduction

The coronavirus disease (COVID-19) has undoubtedly become one of the most memorable events of the year 2020. A major public health issue, COVID-19 is believed to have been first detected in late 2019 in China [[1], [2], [3], [4]]. Due to its massive and progressive spread, in the early 2020 the disease was declared a global pandemic by the World Health Organization [5,6]. The latest updates on COVID-19, dated February 14, 2022, show that there are more than 410 million people around the world who have been infected by this disease, with almost 5.9 million of them having died [7].

To combat the pandemic, a mass vaccination programme has been started globally since 2020. The United Kingdom (UK) has become the first country in the world to administer its citizens a fully tested COVID-19 vaccine on December 8, 2020 [8]. Soon enough, many other countries followed the UK's lead to approve and administer COVID-19 vaccines to their people. As reported by BBC [9] from its ‘Our World in Data’ source [10], the top ten countries with the highest total number of vaccinations carried out to date (February 2022) are China (∼3 billion/B doses), India (∼1.695 B), the United States (∼543 million/M), Brazil (∼369 M), Indonesia (∼323 M), Japan (∼208 M), Pakistan (∼186 M), Vietnam (∼181 M), Mexico (∼169 M), and Germany (∼166 M).

The World Health Organization (WHO) together with Gavi and the Coalition for Epidemic Preparedness Innovations (CEPI), has also promoted the COVAX initiative. As one of the three pillars of the Access to COVID-19 Tools Accelerator – diagnostics, treatments, and vaccines – COVAX has focused on the third one [11,12]. It aims to accelerate the development and manufacture of COVID-19 vaccines and ensure that they are equitably distributed around the world [12,13]. Therefore, with the mass vaccination programme and the COVAX initiative, we may have higher hopes of ending the COVID-19 pandemic sooner and returning to our normal lives before the pandemic occurred.

Despite the future prospects, one simple question remains: Will the mass COVID-19 vaccination programme result in a decrease in confirmed COVID-19 cases? The successful distribution and administration of COVID-19 vaccines across the Globe should decrease the number of COVID-19 confirmed cases. Therefore, to get an insight into this question, in this study we aim to predict and analyse the COVID-19 confirmed cases before and after the mass vaccination programme rolled out. A Deep Learning method, known as the Long Short-Term Memory (LSTM) networks, will be employed as the main prediction method. LSTM was developed to tackle the limitations found in the Recurrent Neural Network (RNN) method, which suffers from short-term memory, vanishing and exploding gradient [14]. As one of the advanced forecasting methods commonly used in time series analysis and other applications with astounding results [15], it could be classified as a black-box model [16].

Various Deep Learning methods, such as Generative Adversarial Networks (GANs), Extreme Learning Machine (ELM), and LSTM, have been employed in COVID-19 research [17] and in spread forecasting for epidemiology [18]. In particular, the LSTM method has been used to predict the COVID-19 epidemic transmission and trends. Chimmula and Zhang [19], for example, developed a forecasting model for the COVID-19 outbreak in Canada by using LSTM networks. With a relatively small amount of data, they predicted that the ending point of COVID-19 outbreak in Canada would be around June 2020 [19]. Wang et al. [20] also used the LSTM method, which was further improved by using a rolling update mechanism and Diffusion Index to predict the COVID-19 epidemic trends in Russia, Peru, and Iran. They predicted that the epidemic in Peru would peak around early December 2020, while the number of positive cases per day in Iran would fall below 1000 by mid-November 2020, in contrast to Russia, which was predicted to have an increment of more than 2000 cases per day by early December 2020 [20]. Another implementation of the LSTM model has been introduced by Pathan et al. [21]. They used LSTM to predict the future mutation rate of SARS-CoV-2, the novel coronavirus that caused the COVID-19 pandemic. They concluded that if more patient data had been made available in an updated time period, the proposed model could be used to predict the mutation rates of this virus on a daily basis [21]. Table 1 summarises several studies that used Machine and Deep Learning methods to predict COVID-19.

**Table 1:** Studies on COVID-19 prediction using Machine and Deep Learning.

Author(s)	Aim(s)	Method(s)	Results
Ribeiro et al. (2020) [22]	To conduct short-term forecasting of COVID-19 cumulative confirmed cases in ten Brazilian states with a high daily incidence.	AutoRegressive Integrated Moving Average (ARIMA), Cubist Regression (CUBIST), Random Forest (RF), Ridge Regression (RIDGE), Support Vector Regression (SVR), Stacking-ensemble Learning	SVR and stacking ensemble are the most suitable tools to forecast COVID-19 cases in the evaluated scenarios.
da Silva et al. (2020) [23]	To forecast the number of COVID-19 new cases in the Brazilian and USA context.	Bayesian Regression Neural Network, Cubist Regression, k-Nearest Neighbors, Quantile Random Forest, and Support Vector Regression + Variational Mode Decomposition (VMD)	VMD-based models are the most suitable tools to forecast COVID-19 cases six days ahead.
Arora et al. (2020) [14]	To predict the number of novel coronavirus (COVID-19) positive reported cases for 32 states and union territories of India.	Deep LSTM, Convolutional LSTM, Bi-directional LSTM (Bi-LSTM)	Based on prediction errors, bi-directional LSTM gives the best results, and convolutional LSTM gives the worst results.
Sinha et al. (2021) [24]	To predict the number of coronavirus confirmed cases for the five topmost affected countries (USA, India, Brazil, Russia, France) across the world.	Artificial Neural Network (ANN), LSTM	LSTM model outperformed the ANN model.
Kuvvetli et al. (2021) [25]	To design a predictive model based on Artificial Neural Network (ANN) model to predict the future number of daily cases and deaths caused by COVID-19 in a generalised way to fit different countries' spreads.	Artificial Neural Network (ANN)	The proposed model could achieve 86% overall accuracy in predicting the mortality rate and 87% in predicting the number of cases.
Verma et al. (2022) [26]	To capture the complex trend of COVID-19 outbreak and perform the forecasting of COVID-19 daily confirmed cases of 7, 14, 21 days for India and its four most affected states (Maharashtra, Kerala, Karnataka, and Tamil Nadu).	Vanilla LSTM, Stacked LSTM, Encoder Decoder-LSTM (ED_LSTM), Bi-LSTM, Convolutional Neural Network (CNN), Hybrid CNN + LSTM	The stacked LSTM and hybrid CNN + LSTM models perform best relative to other models.
Alassafi et al. (2022) [27]	To develop a prediction model for the spread of the COVID-19 outbreak to and throughout Malaysia, Morocco and Saudi Arabia.	RNN, LSTM	The LSTM models showed a 98.58% precision accuracy while the RNN models showed a 93.45% precision accuracy.
Xu et al. (2022) [28]	To predict the number of COVID-19 cases for Brazil, India, and Russia.	CNN, LSTM, CNN-LSTM	The LSTM model had the highest performance.

Although LSTM has been widely used to predict COVID-19 future trends, to the best of our knowledge, this is the first study to apply LSTM networks in the prediction and analysis of COVID-19 confirmed cases before and after the mass vaccination programme was implemented, with a particular focus on the top ten countries with the highest total number of vaccination doses delivered. LSTM was chosen because it has been widely accepted and successfully applied in a variety of cases, particularly in the time series domain. The successful application of the proposed LSTM networks in predicting COVID-19 confirmed cases before and after the mass vaccination programme was implemented could aid decision-makers in devising better pandemic management strategies.

Artificial intelligence (AI) is a new paradigm for healthcare systems, and it is important to note that intelligent machine learning algorithms can be used to analyse COVID-19 data and provide information for decision-making processes. This implies that tools powered by AI can aid in predicting the number of confirmed COVID-19 cases. A fundamental requirement is the availability of sufficient data to train the respective models. Earlier in the pandemic, the majority of AI-powered tools utilised by previous studies to forecast and predict the pandemic were limited to proof-of-concept models. However, as more and more data are generated every day, this presents the opportunity to reevaluate the robustness of existing algorithms.

A large number of algorithms are created frequently. And while we recognise the importance of developing new and perhaps better algorithms, it is also important that we maintain a balance by utilising what we already have that has been proven effective. In this regard, then, our work contributes to the existing body of knowledge. The fact that we use a well-known method with a track record of proven robustness (i.e., LSTM) to predict the number of confirmed COVID-19 cases and the future trend is an advantage that helps to counteract the phenomenon known as COVID-19, which is still poorly understood. Overall, we were able to demonstrate that the mass vaccination programme contributes to reducing and controlling the spread of the COVID-19 disease in those countries, as indicated by the decreasing future trends after the mass vaccination programme was administered. This can help relevant decision-makers make better practical decisions and take appropriate actions or measures to contain or limit the coronavirus's spread.

## 2 Materials and methods

In this section, we first describe the data source being used in this study. Next, we explain the basic concept of LSTM networks that are used as the main prediction method in this study, followed by a brief explanation of several evaluation metrics used.

### 2.1 Data source

The main data source of COVID-19 confirmed cases used in this study was collected from a GitHub data repository, which is operated and maintained by the Johns Hopkins University (JHU) Center for Systems Science and Engineering [29]. This repository is updated and curated by a team of scientists at JHU since the early time of COVID-19 outbreak, and the data visualisation is depicted in an online real-time interactive dashboard [30]. It can be accessed publicly and has been widely used in many publications [16,19,20].

We used the global time series data of COVID-19 confirmed cases, which was named as ‘time_series_covid19_confirmed_global.csv’ and taken on February 14, 2022 (last recorded data on February 12, 2022). The document consists of more than 280 regions' data, but we will focus on the ten countries or regions with the highest total vaccinations volume up to date [9,10], namely China, India, the United States, Brazil, Indonesia, Japan, Pakistan, Vietnam, Mexico, and Germany. Table 2 presents the summary statistics of the dataset used in this study.

**Table 2:** Statistics summary for ten countries with the highest total vaccinations of COVID-19.

Summary	Count	Mean	Std	Min	25%	50%	75%	Max
China	753	95455.5	17542.08	548	86,990	100,127	105,902	123,728
India	753	15,902,771	14,156,129	0	1,531,669	10,766,245	31,969,954	42,631,421
United States	753	23,837,230	20,024,848	1	4,346,567	26,470,178	35,905,164	77,707,349
Brazil	753	10,816,879	8,614,708	0	2,503,681	9,237,011	20,169,989	27,434,286
Indonesia	753	1,640,969	1,676,288	0	102,051	1,089,308	3,666,031	4,763,252
Japan	753	647701.8	753700.2	2	32,092	392,533	1,033,214	3,842,551
Pakistan	753	629330.3	467572.2	0	275,225	547,648	1,071,620	1,483,798
Vietnam	753	301,036	596570.7	0	446	1850	215,560	2,484,481
Mexico	753	1,826,320	1,479,265	0	402,697	1,869,708	2,971,817	5,283,852
Germany	753	2,514,732	2,534,181	0	207,707	2,232,327	3,797,849	12,391,463

### 2.2 LSTM networks

**Fig. 1:**
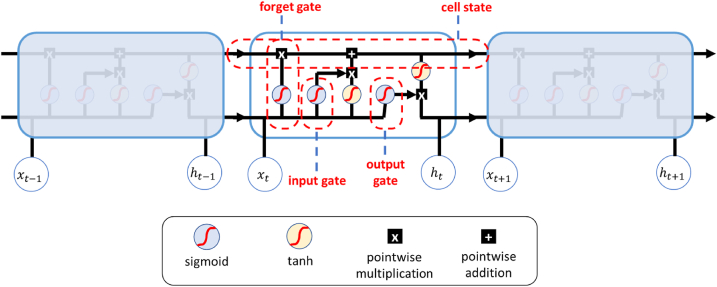
An LSTM cell and its gates [35].

Long Short-Term Memory (LSTM) is an advanced soft computing method, which was derived from the Recurrent Neural Network (RNN). RNN itself actually is one of the many types of Artificial Neural Networks (ANN) methods, which was proposed to overcome the ANN's disadvantage in handling the time correlation in data sequence. It adds canonical connections to neurons in the networks, so that the sequence-to-sequence mapping between input and output data can be built by RNN [31]. Unfortunately, classical RNN still struggles with the long-range dependencies, suffering from exploding gradient or, in contrast, from vanishing gradient, which limits its ability to learn the long-term temporal correlations [32]. Therefore, LSTM was introduced by Hochreiter and Schmidhuber (1997) to overcome this limitation by using memory cells [33]. These cells are self-connected and store the networks' temporal state by using a three-gate mechanism, composed of the input gate, the output gate, and the forget gate [33]. Fig. 1 depicts an LSTM cell, which contains all those three gates and the cell state [34,35].

LSTM gates are simply used as a way to control how much information can be passed. Commonly, they are composed of a sigmoid neural network layer and a pointwise multiplication operation. Forget gate is used to forget the information in the cell state selectively, while the input gate is used to determine what new information will be stored in the current cell state. Lastly, the output gate is used to find what value we want to output [31].

The first part of the LSTM cell is the forget gate. It is used to control the magnitude to forget the hidden state of the previous cell and it can be expressed as shown in Eq 1:

(1) $$f_{t}=\sigma \left(W_{f}h_{t-1}+U_{f}x_{t}+b_{f}\right)\text{,}$$

where $f_{t}$ denotes the forget gate value at the current cell, which ranges from 0 (completely forget) to 1 (completely keep), and $W_{f},U_{f}$ are the weights of the networks; $b_{f}$ is the bias variable value, $h_{t-1}$ is the prior hidden state value, and $x_{t}$ is the new input value at the current cell.

Next, to update the cell state, we use the input gate. There are two actions that will be taken in this step. First, for the input gate, we pass the prior hidden state value ($h_{t-1}$) and the current input value ($x_{t}$) into a sigmoid function as shown in Eq [2]. The resulting value of the input gate ($i_{t}$) decides the magnitude of the new information that will be kept in the current cell, where 0 means ‘completely ignore’ and 1 means ‘completely keep’. Second, we also pass the prior hidden state value ($h_{t-1}$) and the current input value ($x_{t}$) into the tanh function to help regulate the network as shown in Eq [3]. Similarly, when it comes to the forget gate, there are some weights of the networks and bias values involved in this step, as denoted by $W_{i},W_{C},U_{i},U_{C},b_{i}\text{,}$ and $b_{C}$.

(2) $$i_{t}=\sigma \left(W_{i}h_{t-1}+U_{i}x_{t}+b_{i}\right)\text{,}$$

(3) $${\tilde{C}}_{t}=\tanh\left(W_{C}h_{t-1}+U_{C}x_{t}+b_{C}\right)\text{.}$$

At this point, we have enough information to calculate the (current) cell state ($C_{t}$). The previous cell state ($C_{t-1}$) will be pointwise multiplied with the forget vector ($f_{t}$). Then, we do a pointwise addition with the output from the input gate ($i_{t}$), which has been pointwise multiplied with the cell candidate value (${\tilde{C}}_{t}$), as shown in Eq [4].:

(4) $$C_{t}=f_{t}\;⨀\;C_{t-1}+i_{t}\;⨀\;{\tilde{C}}_{t}\text{.}$$

In the last step, we use the output gate to decide what the next hidden state should be (*i.e*., the current hidden state value, $h_{t}$). First, we pass the prior hidden state value ($h_{t-1}$) and the current input value ($x_{t}$) into the sigmoid function as shown in Eq [5]. Here, $W_{o},U_{o}\text{,}$ and $b_{o}$ are the corresponding networks weights and bias values for the output gate. Then, we pass the newly found cell state ($C_{t}$) to the tanh function and pointwise multiply the output with the sigmoid output from the output gate ($o_{t}$) as shown in Eq [6]. The output from this last process is the current hidden state value ($h_{t}$), which will be passed together with the current cell state ($C_{t}$) to the next time step.

(5) $$o_{t}=\sigma \left(W_{o}h_{t-1}+U_{o}x_{t}+b_{o}\right)$$

(6) $$h_{t}=o_{t}\;⨀\;\tanh\left(C_{t}\right)$$

### 2.3 Evaluation metrics

Three different prediction error criteria will be used as the evaluation metrics in this study. These are the Mean Absolute Error (MAE), the Root Mean Squared Error (RMSE), and the Mean Absolute Percentage Error (MAPE). The first two show the degree of error in a unit value, while the last one shows the degree of error in a percentage value. As described by Shahid et al. [33] and Hansun et al. [36,37], all those three criteria can be expressed as shown in Eq. [7], Eq. [8], and Eq. [9], respectively:

(7) $$MAE=\frac{1}{n}\sum_{t=1}^{n}\left|Y_{t}-F_{t}\right|\text{,}$$

(8) $$RMSE=\sqrt{\frac{1}{n}\sum_{t=1}^{n}{\left(Y_{t}-F_{t}\right)}^{2}}\text{,}$$

(9) $$MAPE=\left(\frac{1}{n}\sum_{t=1}^{n}\left|\frac{Y_{t}-F_{t}}{Y_{t}}\right|\right)∙100\%\text{,}$$

where n is the total number of data, $Y_{t}$ is the actual value, and $F_{t}$ is the predicted value. Moreover, we will also use the popular Mean Squared Error (MSE) criterion in the calculation of the loss function during the LSTM networks training. The formula for MSE is shown in Eq10 [38].

(10) $$MSE={\left(RMSE\right)}^{2}=\frac{1}{n}\sum_{t=1}^{n}{\left(Y_{t}-F_{t}\right)}^{2}\text{.}$$

## 3 Results and discussion

In this section, first we explain the data splitting and the pre-processing of the ten countries considered in this study before we move to the implementation and prediction results of COVID-19 confirmed cases by using LSTM networks. The analysis and discussion of the effect of the mass vaccination programme in these countries will be provided in the last part of this section.

### 3.1 Data splitting, pre-processing, and model building

**Fig. 2:**
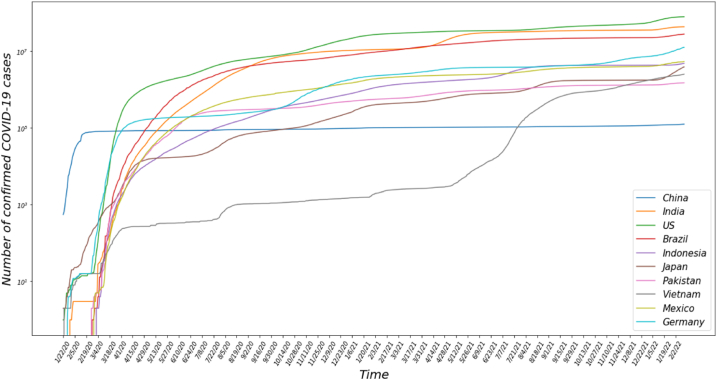
Confirmed COVID-19 cases in the top ten countries with the highest vaccination doses (shown in a log scale).

There are ten countries considered in this study and these are those that have the highest total number of vaccinations [9,10]: China, India, the United States, Brazil, Indonesia, Japan, Pakistan, Vietnam, Mexico, and Germany. First, we will plot the number of COVID-19 confirmed cases in these countries from the first available data on January 22, 2020 to the last recorded data on February 12, 2022 as shown in Fig. 2. We will consider those data as the ‘All Time’ data that show the number of COVID-19 confirmed cases for each country even after the mass vaccination programme started. Next, for simplicity, we assume that all countries considered have the same effective date for the mass vaccination programme having taken effect in controlling the disease, *i.e*., on February 1, 2021. Therefore, the available data for each country since January 22, 2020 until February 1, 2021 will be considered as the ‘Before Vaccination’ data that show the number of COVID-19 confirmed cases before the mass vaccination programme had taken effect in those countries.

Next, for the data splitting process, we use the 80:20 ratio to split the data of each considered country into training and test set. Moreover, we also use 14 timestamps (span), meaning that the 14 last days will be used to consider the future number of COVID-19 confirmed cases for each country. Table 3 shows the splitting numbers of each country's data considered in this study.

**Table 3:** Data splitting for each country.

No	Country	All Time Data (days)			Before Vaccination Data (days)		
		All Data	Train	Test	All Data	Train	Test
1	China	753	602	151	376	300	76
2	India	745	595	150	368	294	74
3	United States	753	602	151	376	300	76
4	Brazil	718	574	144	341	272	69
5	Indonesia	713	570	143	336	268	68
6	Japan	753	602	151	376	300	76
7	Pakistan	719	575	144	342	273	69
8	Vietnam	752	601	151	375	299	76
9	Mexico	716	572	144	339	271	68
10	Germany	748	598	150	371	296	75

After the data splitting process, we conduct the data normalisation process (feature scaling) by using the MinMaxScaler transformation method. Then, to incorporate the defined timestamps, we create a new function called ‘create_dataset’. Lastly, we convert the data shape into a 3D array shape, which is the accepted data shape by the LSTM model in the Keras library.

For the model building process, we use a well-known Deep Learning package for Python, namely Keras. It runs on top of the TensorFlow Machine Learning platform. Several modules from Keras are used to build the LSTM networks in this study, *i.e*., ‘Sequential’ to initialise the neural networks, ‘Dense’ to add a densely connected neural network layer, ‘LSTM’ to add the Long Short-Term Memory layer, and ‘Dropout’ to add a dropout layer to prevent overfitting. In summary, we build five-layer neural networks comprised of two LSTM layers, two Dropout layers, and one Dense (output) layer. Interested readers may find the source code and data used in this study in the GitHub repository at https://github.com/senghansun/COVID-19-with-LSTM.

### 3.2 Prediction results and analysis

**Fig. 3:**
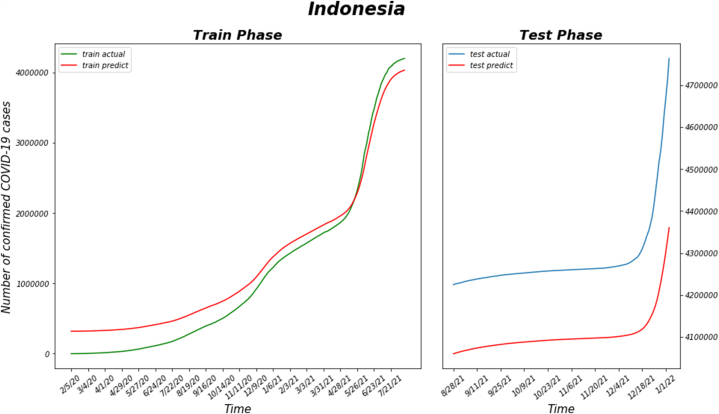
Prediction results for Indonesia based on ‘All Time’ data (left panel shows the actual and predicted ‘training’ data, right panel shows the actual and predicted ‘test’ data).

**Fig. 4:**
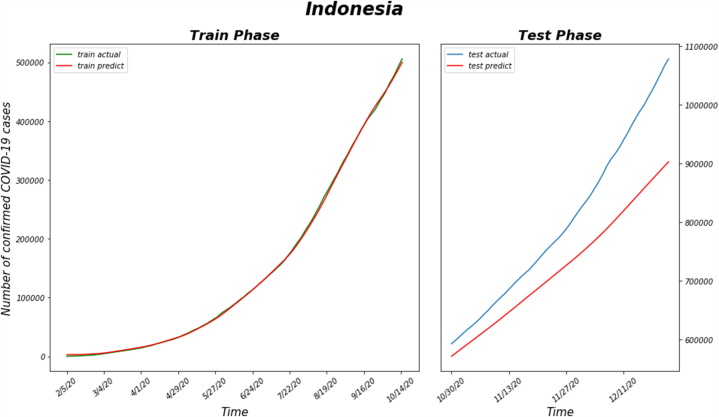
Prediction results for Indonesia based on ‘Before Vaccination’ data (left panel shows the actual and predicted ‘training’ data, right panel shows the actual and predicted ‘test’ data).

In this section, we describe the prediction results of COVID-19 confirmed cases for all the ten countries of interest by using a Deep Learning method, namely, the LSTM networks. We divide the prediction results into two categories, one for the ‘All Time’ data and another one for the ‘Before Vaccination’ data. ‘All Time’ results show the prediction results since the first available data to the last recorded data of each country after the mass vaccination programme started. Meanwhile, ‘Before Vaccination’ results show the prediction results since the first available data for each country to the cut date when we assumed the mass vaccination programme should have taken effect, *i.e*., on February 1, 2021. Fig. 3, Fig. 4 show the prediction results for Indonesia of both categories, respectively. Meanwhile, the complete prediction results for all countries are provided in the supplementary file (**S1**).

As previously stated, we also evaluate the prediction results of COVID-19 confirmed cases for each country by using three different evaluation metrics, namely, MAE, RMSE, and MAPE. Table 4 shows the corresponding evaluation metrics results on the test set for each country.

**Table 4:** Evaluation metrics results for the proposed LSTM networks.

No	Country	MAE	RMSE	MAPE (%)	MAE	RMSE	MAPE (%)
		All Time			Before Vaccination		
1	China	5481.892	5888.154	4.776	3968.679	4039.007	4.109
2	India	2534234.563	2797838.637	7.322	54341.213	58747.140	0.537
3	United States	4967555.618	5516520.691	8.720	2937303.661	3185617.821	13.782
4	Brazil	1597245.739	1643210.711	6.958	267944.556	303655.959	3.263
5	Indonesia	180809.188	186428.061	4.200	75618.777	88394.719	8.767
6	Japan	74402.208	174840.944	2.646	58662.687	69678.143	20.722
7	Pakistan	19910.454	20466.925	1.518	28230.114	28386.097	5.748
8	Vietnam	261791.011	360565.827	15.083	43.566	63.216	2.809
9	Mexico	79458.387	84013.698	1.987	157632.919	169329.206	10.178
10	Germany	440082.578	569720.458	6.558	614326.058	661147.047	33.965

As it can be inferred from Table 4 and from the prediction results plots in Fig. 3, Fig. 4, LSTM networks could predict the number of COVID-19 confirmed cases quite well. LSTM networks work particularly well especially in the cases of Pakistan, Mexico, and Japan for ‘All Time’ scenario, and India, Vietnam, and Brazil for ‘Before Vaccination’ scenario, for which the lowest prediction error results based on the evaluation metrics are achieved. In the regression task, lower MAE, RMSE, and MAPE values indicate higher prediction accuracy [39,40]. Furthermore, the average MAPE scores for ‘All Time’ versus ‘Before Vaccination’ are 5.977% and 10.388%. Hence, the prediction results using ‘All Time’ data scenario have better accuracy level, mostly impacted by higher number of time series data available to be used in the model development.

We also compare the prediction results obtained with LSTM networks to those obtained with another Deep Learning method, the Vanilla Recurrent Neural Network (Vanilla RNN), which serves as the benchmark method. For the Vanilla RNN, a similar five-layer architecture was used, and the results were evaluated using MAE, RMSE, and MAPE. Table 5 displays the Vanilla RNN evaluation results using the same dataset.

**Table 5:** Evaluation metrics results for Vanilla RNN.

No	Country	MAE	RMSE	MAPE (%)	MAE	RMSE	MAPE (%)
		All Time			Before Vaccination		
1	China	4269.936	5160.330	3.681	8207.455	8314.650	8.560
2	India	1701323.469	1867145.899	4.911	1379079.409	1387292.461	13.401
3	United States	5306281.659	6194355.225	9.095	7834138.913	8156805.251	37.718
4	Brazil	931029.183	1067898.511	3.978	885525.878	931792.401	10.976
5	Indonesia	130267.288	133373.318	3.055	106484.409	127270.603	12.224
6	Japan	145128.987	218623.615	6.439	126724.599	138050.298	47.809
7	Pakistan	8763.209	15148.221	0.631	64313.161	65504.417	12.999
8	Vietnam	552050.702	681562.949	33.234	167.299	171.886	11.220
9	Mexico	74985.719	96466.140	1.715	358630.341	364609.578	23.762
10	Germany	900887.739	1310741.397	10.985	269397.174	309302.287	14.385

In general, Vanilla RNN may provide lower MAE and RMSE scores than LSTM in both the ‘All Time’ and ‘Before Vaccination’ scenarios. It does, however, provide a much higher MAPE score than LSTM networks. Using Vanilla RNN, the average MAPE scores for ‘All Time’ and ‘Before Vaccination’ are 7.772% and 19.305%, respectively. Again, the prediction results based on ‘All Time’ data have better accuracy level than the ‘Before Vaccination’ scenario. When the average MAPE scores for LSTM and Vanilla RNN are compared, it is clear that LSTM has lower MAPE scores for both the ‘All Time’ and ‘Before Vaccination’ scenarios than Vanilla RNN (5.977% vs 7.772% and 10.388% vs 19.305%, respectively). As a result, it is possible to conclude that the proposed LSTM networks outperform the Vanilla RNN in terms of MAPE.

Moreover, we also tried to find the future trend projection of COVID-19 confirmed cases in each country considered by comparing the future prediction result with the last known data record (one period ahead). Table 6 shows the future prediction as well as the trend percentage for each country both for the ‘All Time’ and ‘Before Vaccination’ data using the proposed LSTM networks.

**Table 6:** Future trend prediction.

No	Country	Future Prediction	Trend Percentage	Future Prediction	Trend Percentage
		All Time		Before Vaccination	
1	China	112,489	↓ −9.083%	114,413	↓ −7.528%
2	India	42,734,970	↑ 0.243%	41,958,874	↓ −1.578%
3	United States	69,443,021	↓ −10.635%	69,194,058	↓ −10.956%
4	Brazil	25,035,506	↓ −8.744%	26,606,945	↓ −3.016%
5	Indonesia	4,360,166	↓ −8.462%	4,660,538	↓ −2.156%
6	Japan	3,227,078	↓ −16.017%	2,838,903	↓ −26.119%
7	Pakistan	1,465,844	↓ −1.210%	1,428,577	↓ −3.722%
8	Vietnam	1,605,154	↓ −35.393%	1,422,270	↓ −42.754%
9	Mexico	5,288,044	↑ 0.079%	4,777,232	↓ −9.588%
10	Germany	10,336,484	↓ −16.584%	16,443,102	↑ 32.697%

Based on the prediction results, most countries in both categories have a downward trend for the number of COVID-19 confirmed cases. The only two exceptions are posed by India with an upward trend of +0.243% and Mexico of +0.079% for the ‘All Time’ data; while for the ‘Before Vaccination’ data, the only exception is posed by Germany (+32.697%). However, as we can see from the results, the mass vaccination programme could help in controlling the pandemic, even for those countries that have increased trend projections. The prediction is that Germany, for example, will have quite a big increasing number of confirmed cases in the future if they do not start the mass vaccination programme (#16, 443, 102, +32.697%), while they are projected to have a downward trend when they have started the mass vaccination programme (#10, 336, 484, −16.584%).

In general, the mass vaccination programme has a positive effect in terms of contributing to decreasing and controlling the spread of the COVID-19 disease in most countries considered. We can easily see that by comparing the future trend results for each country, both for ‘All Time’ (when the mass vaccination programme has been started and considered to have taken effect) and ‘Before Vaccination’ data. However, out of the ten countries considered, two of them have shown slightly different results. Both India and Mexico have a greater decreasing trend for ‘Before Vaccination’ than for ‘All Time’ data, which means that the mass vaccination programme seems not to be showing any better effect than if the programme had not been administered. This finding might be rooted in several causes, such as the slow government response to handle the pandemic on the early date, the improper handling of the mass vaccination programme by related stakeholders, the increasing in testing capacity on the recent date, and even the lack of community support for the success of the mass vaccination programme. Moreover, it is worth noticing that a new COVID-19 variant, named Omicron, has emerged since November 2021 [41] and affected a great number of people worldwide. It even predicted to be the root of the next wave of COVID-19 outbreak in several countries [42,43]. Without the mass vaccination program, a more severe catastrophe caused by the disease may happen.

## 4 Conclusions

The Coronavirus Disease 2019 (COVID-19) has struck us for more than two years since it was declared a global pandemic by the World Health Organization (WHO) in March 2020 [5]. It has affected every aspect of our lives, with more than 410 million people around the world having been infected by this disease and almost 5.9 million of them having died (as of February 14, 2022). As a response to this major public health threat, the mass vaccination programme has been started and administered in many countries around the world since the end of 2020.

In this study, we have aimed to investigate whether the COVID-19 mass vaccination programme really works in terms of contributing to decreasing and controlling the spread of the COVID-19 disease. Therefore, we tried to predict the future number and trend of COVID-19 confirmed cases for the ten countries with the highest number of vaccinations to date, namely, China, India, the United States, Brazil, Indonesia, Japan, Pakistan, Vietnam, Mexico, and Germany. We grouped the recorded data into two categories, *i.e*., the ‘All Time’ data and the ‘Before Vaccination’ data. Then, by using a well-known Deep Learning algorithm, *i.e.*, the Long Short-Term Memory (LSTM) networks, we built a model for each category of data and used them to predict the future number and trend of COVID-19 confirmed cases for each country.

Based on the experimental results, we found that the LSTM networks model could be used to predict the future number and trend of COVID-19 confirmed cases quite well in most countries considered. The average MAPE scores for ‘All Time’ versus ‘Before Vaccination’ scenarios are 5.977% and 10.388% respectively. We also found that the mass vaccination programme has a positive effect in terms of contributing to decreasing and controlling the spread of COVID-19 disease in those countries. The only exception is represented by India and Mexico, with both countries having a greater decreasing trend when we predicted the data using the ‘Before Vaccination’ model. Some factors might cause this finding, such as the slow government response, the improper handling and administration of the mass vaccination programme, the increasing tracing number, the lack of community support for the success of this programme, and even the impact of the new COVID-19 variant named Omicron. Future studies on the barriers to the mass vaccination programme could be taken to answer this question and correlate this finding in more detail.

There are several limitations in our study. Firstly, we applied relatively simple five-layers LSTM networks in predicting the future confirmed cases of COVID-19. We did not put our focus in the optimization and introduction of a new and better prediction model, but rather on the applicability of a well-known Deep Learning method, i.e., the LSTM networks, in predicting confirmed cases ‘before’ and ‘after’ the mass vaccination programme rollout. Another limitation is on performance metrics used in this study. We only use three popular error measurement criteria, namely MAE, RMSE, and MAPE, which could not directly measure the trend movement from the prediction results. Directional Statistics (DS) as has been used in several studies [39,40] can be used to assess this trend more accurately.

Given that the future is unpredictable, present predictions must be viewed critically. Nonetheless, a more precise estimate of the number of confirmed COVID-19 cases is essential for optimising available resources and slowing or stopping the pandemic's progression. In addition, our findings can be used to encourage the general public to consider and adhere to the vaccination measures mandated by local and national authorities to halt the pandemic's spread. In this regard, we hope that the present paper can aid a variety of stakeholders in their decision-making processes, thereby facilitating the implementation of appropriate measures to prevent the spread of COVID-19. Overall, this has significant implications for practice, as it would allow policymakers and healthcare providers to plan and determine where to deploy resources.

## Declarations

### Ethics approval and consent to participate

Not applicable.

### Consent for publication

Not applicable.

### Availability of data and materials

The dataset used and/or analyzed during the study together with the code are available at https://github.com/senghansun/COVID-19-with-LSTM.

### Author contribution statement

Seng Hansun: Conceived and designed the experiments; Performed the experiments; Analyzed and interpreted the data; Contributed reagents, materials, analysis tools or data; Wrote the paper. Vincent Charles; Tatiana Gherman: Analyzed and interpreted the data; Contributed reagents, materials, analysis tools or data; Wrote the paper.

### Funding statement

This research did not receive any specific grant from funding agencies in the public, commercial, or not-for-profit sectors.

### Data availability statement

Data associated with this study has been deposited at https://github.com/senghansun/COVID-19-with-LSTM.

### Declaration of interest's statement

The authors declare no conflict of interest.
