# Supplementary figures and images for: The role of the mass vaccination programme in combating the COVID-19 pandemic: An LSTM-based analysis of COVID-19 confirmed cases
Source: Heliyon. 2023 Mar 8;9(3):e14397. doi: 10.1016/j.heliyon.2023.e14397 (PMC9993739; doi:10.1016/j.heliyon.2023.e14397)

**Supplementary File: S1 Prediction Results**

**‘All Time’ data**


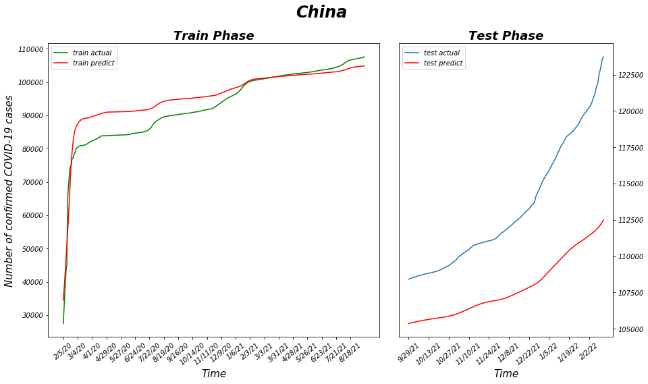


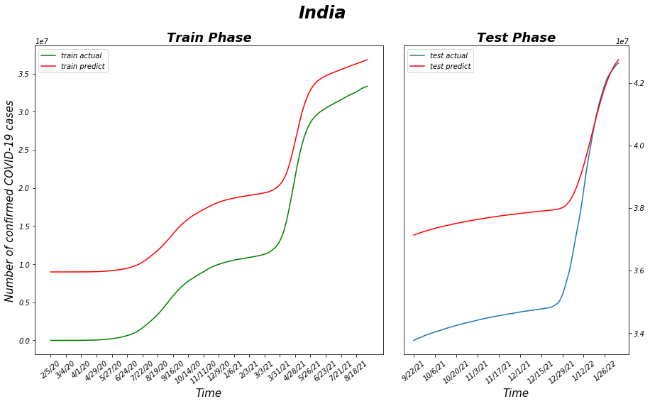


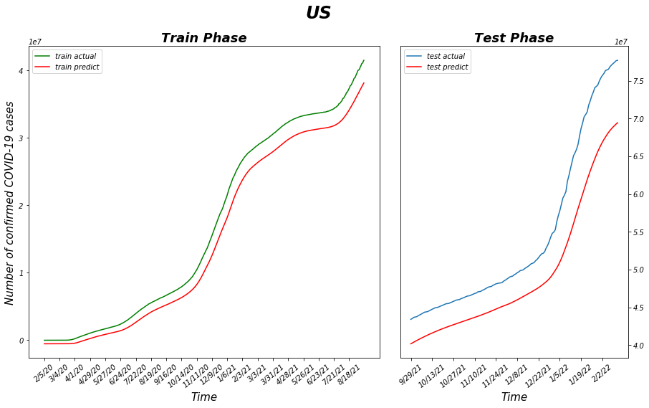


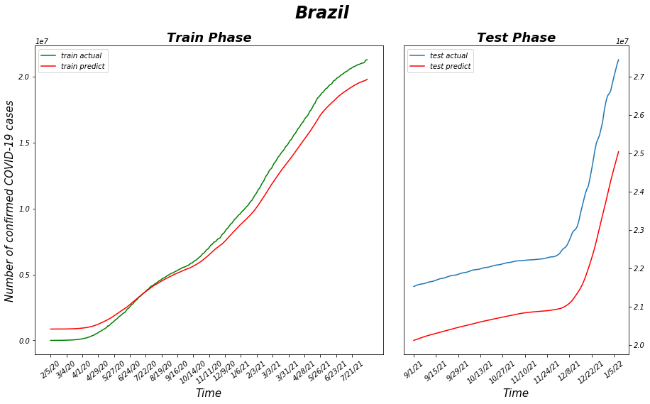


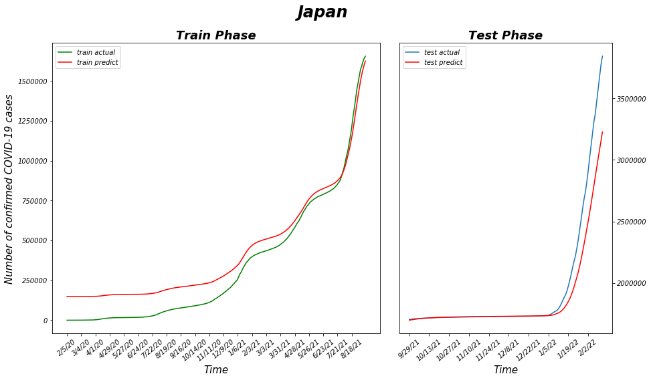


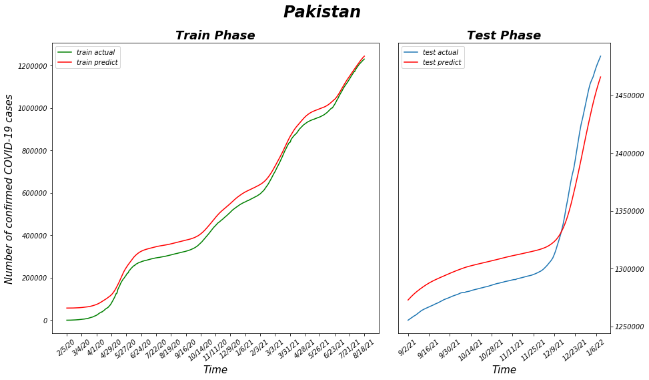


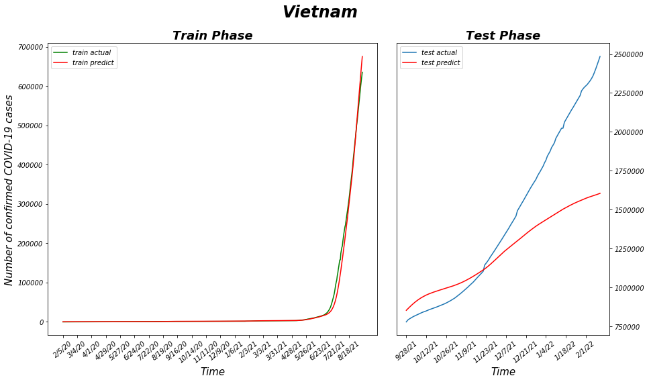


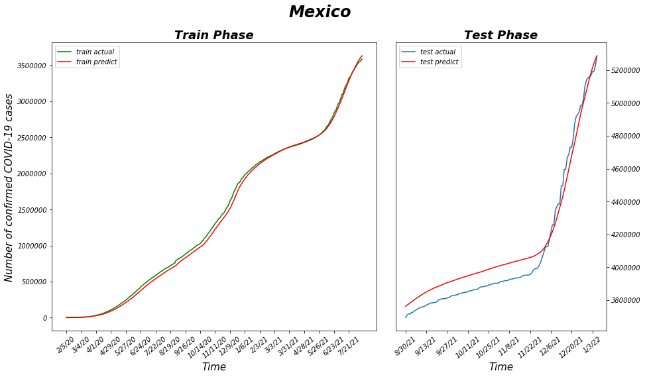


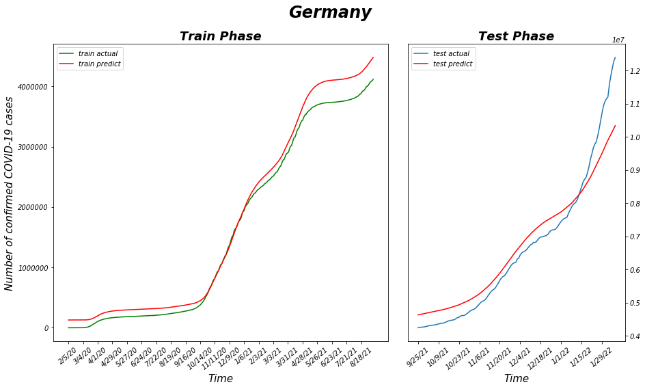


**‘Before Vaccination’ data**


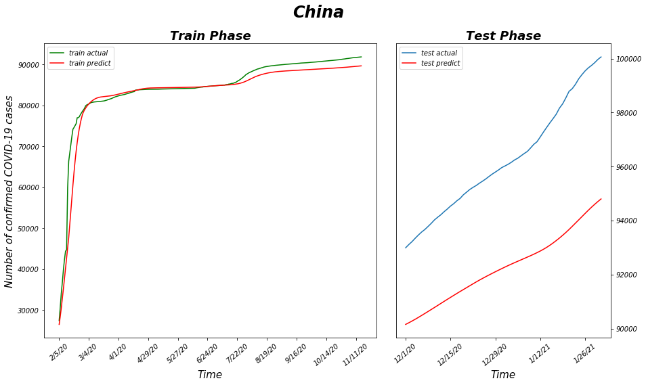


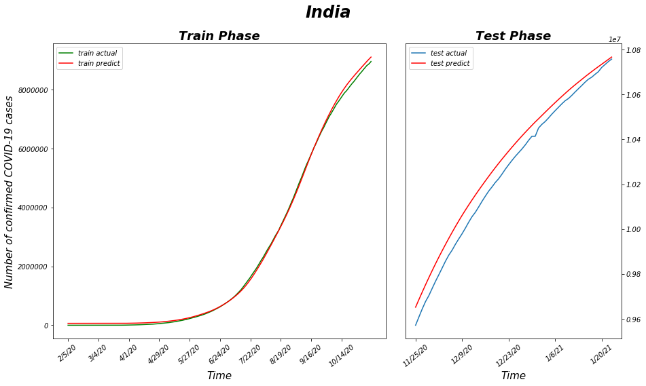


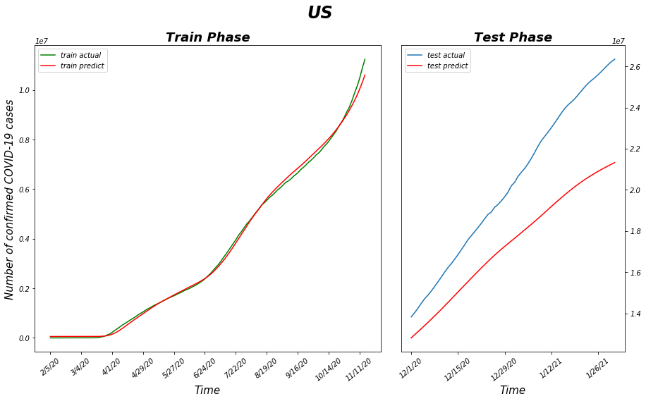


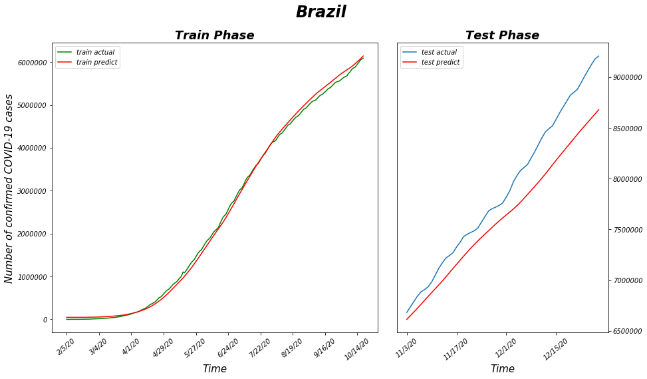


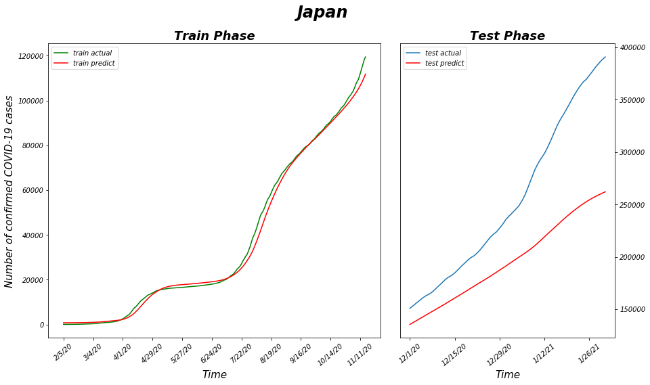


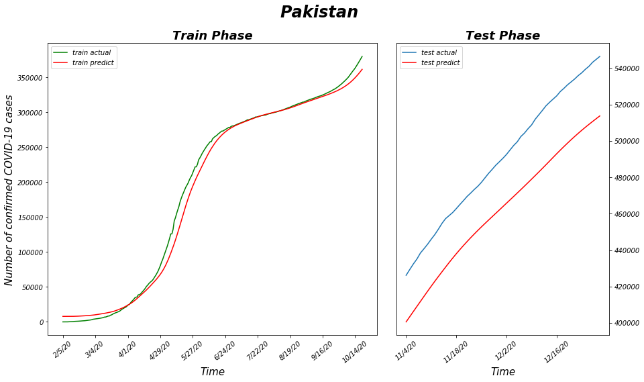


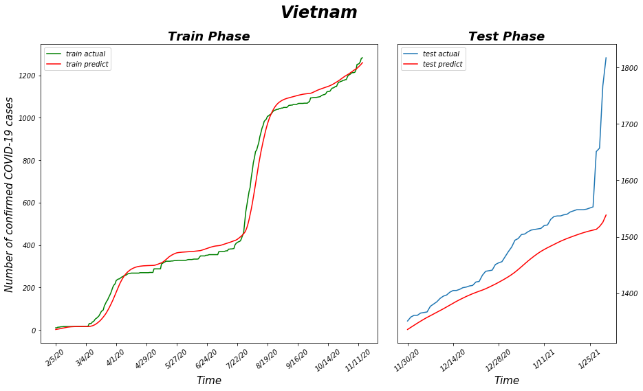


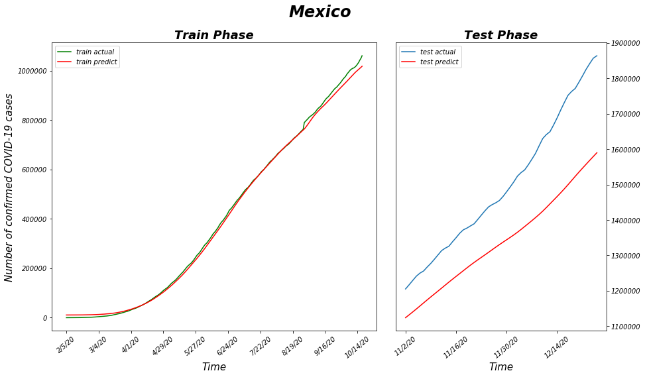


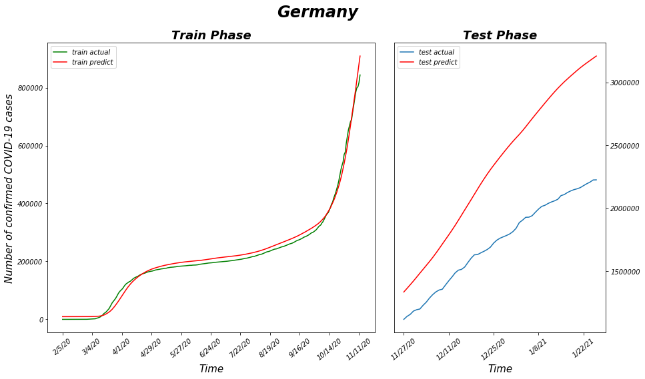

Supplement: Multimedia component 1 [file mmc1.docx]
